# Validation of salivary uric acid remote self-monitoring for early prediction of hypertensive disorders of pregnancy: study protocol for a prospective, observational, multicentre cohort study

**DOI:** 10.1136/bmjopen-2024-094421

**Published:** 2025-06-18

**Authors:** Basia Chmielewska, Isabel Reading, Amarnath Bhide, Ramesh Ganapathy, Baskaran Thilaganathan

**Affiliations:** 1 Department of Obstetrics and Gynaecology, St George’s University Hospitals NHS Foundation Trust, London, UK; 2 St George’s University of London, London, UK; 3 University of Southampton, Southampton, UK; 4 Department of Obstetrics and Gynaecology, Epsom and Saint Helier University Hospitals NHS Trust, Carshalton, UK

**Keywords:** Hypertension, Pregnancy, Pregnant Women, OBSTETRICS, Maternal medicine

## Abstract

**Introduction:**

Hypertensive disorders of pregnancy (HDP), including gestational hypertension and pre-eclampsia, affect approximately 10% of pregnancies worldwide and contribute significantly to fetal and maternal morbidity and mortality. Early identification of HDP would facilitate targeted surveillance and personalised care in order to mitigate the severity of complications and improve pregnancy outcomes. Uric acid is a marker of oxidative stress, inflammation and endothelial dysfunction, and has been proposed as a predictor of hypertensive disease. Salurate is a salivary uric acid test that has the potential to identify pregnant women at risk of developing HDP several weeks before clinical manifestation.

**Methods and analysis:**

This is a prospective, multicentre, observational, cohort study with health economics evaluation. Women aged 16 and above, with a viable singleton pregnancy at <16 weeks gestation and a combined first trimester pre-eclampsia risk of >1:300 will be eligible for recruitment. Participants will perform weekly remote salivary uric acid testing from enrolment until the conclusion of pregnancy and upload results of colourimetric paper tests via a smartphone application. We will validate a predictive algorithm that analyses colour data from several consecutive samples to detect patterns that predict whether HDP is likely to occur. The primary outcome is test performance for the prediction of HDP. Secondary outcomes include adherence to sampling and test performance for predicting gestational diabetes, stillbirth and fetal growth restriction. Data on pregnancy outcomes will be collected from the medical notes, compared with the predictions made by the algorithm and subjected to statistical analysis.

**Ethics and dissemination:**

Approval has been obtained from Cambridge East Research Ethics Committee (REC reference 24/EE/0123), Medicines and Healthcare products Regulatory Agency (CI/2024/0038/GB) and Health Research Authority (IRAS ID 337290). Results of the study will be published in peer-reviewed journals and presented at national and international conferences.

**Trial registration number:**

ISRCTN17992452.

**Protocol version:**

4, 4 July 2024.

**STRENGTHS AND LIMITATIONS OF THIS STUDY**

- This is a large, multicentre study which aims to include participants from a broad range of ethnicities and socioeconomic backgrounds.
- The secondary outcomes will allow us to assess if salivary uric acid could also be used as a predictor of related placentally mediated adverse pregnancy outcomes.
- The potential for inadequate assessment of specificity or sensitivity of salivary uric acid for hypertensive disorders of pregnancy is mitigated by employing a large sample size.
- Possible poor compliance with a weekly testing regime will be partly mitigated by reminders through a customised mobile application and opportunities to make up missed tests.

## Introduction

 Hypertensive disorders of pregnancy (HDP) affect approximately 10% of pregnancies worldwide and are among the leading causes of maternal and fetal morbidity and mortality, contributing to approximately 14% of maternal deaths every year.^1 2^ Early prediction and diagnosis would facilitate targeted surveillance, which would improve allocation of resources for healthcare providers and avoid unnecessary hospital attendance for patients. Traditional antenatal surveillance includes blood pressure and urinalysis at every visit, which identifies HDP at the time of manifestation.^3^ Recent advancements in first-trimester combined screening, such as with the Fetal Medicine Foundation (FMF) algorithm, have improved risk assessment for the development of pre-eclampsia, which facilitates effective preventative interventions such as daily aspirin from the first trimester.^4^ The introduction of soluble fms-like tyrosine kinase-1 and placental growth factor (PlGF) as diagnostic triage tools in symptomatic women has aided clinical decision making in admission and treatment of women with suspected HDP.^5^,^7^ There remains no effective tool that aids continuous and dynamic risk assessment of HDP throughout pregnancy in asymptomatic women, which would allow for a personalised care plan and improved mitigation of the severity of disease and complications.

Uric acid has been implicated in oxidative stress, inflammation and endothelial dysfunction and, therefore, proposed as a useful biomarker and treatment target in neurological, cardiovascular and metabolic conditions.^8^ ^9^ In pregnancy, uric acid has been proposed as a pathogenic, diagnostic and predictive marker of hypertensive disease, with some studies suggesting salivary uric acid could be used as a surrogate, or even more accurate, marker in hypertensive diseases in pregnancy.^10 11^ 12,14 Salivary biomarkers for disease detection and monitoring are a potential tool for a variety of conditions.^15^ Saliva sampling is simple, non-invasive and does not require clinical involvement, making it ideal for remote monitoring. Pilot studies have demonstrated that elevated salivary uric acid in pregnant women correlates with an increased risk of developing HDP.^13^

The Salurate system is a weekly salivary uric acid self-sampling test with the potential to provide predictive information before symptoms appear. It could fill a critical gap in current clinical approaches, allowing for proactive and timely management. If the study demonstrates Salurate’s predictive capabilities are reliable and accurate, its implementation could lead to improved maternal and fetal health outcomes. Early detection of high-risk pregnancies may enable healthcare providers to initiate appropriate interventions promptly, reducing the incidence and severity of HDP, and ultimately leading to improved maternal and neonatal health. Salurate has the benefits of being convenient, simple, pain-free, home-use and non-invasive. As a remote monitoring tool, Salurate effectively minimises the burden of hospital visits for expectant mothers and fosters seamless communication between women and healthcare professionals.

The Salurate study will add to the expanding pool of evidence supporting the adoption of technology-driven healthcare solutions, especially in obstetrics. The findings from this study have the potential to influence clinical practice and public health policies. If Salurate proves to be effective in predicting HDP, it could be integrated into routine prenatal care protocols, enabling healthcare providers to offer more personalised and proactive care to pregnant women.

### Objectives

To assess the efficacy of the Salurate pregnancy remote self-monitoring system in predicting the onset of HDP. By comparing the Salurate system with traditional screening and diagnostic methods, this study aims to determine the sensitivity, specificity, positive predictive value (PPV) and negative predictive value (NPV) of Salurate in identifying pregnant women who will go on to develop HDP. Additionally, the study aims to evaluate the time-to-diagnosis using the Salurate system in comparison to conventional diagnostic approaches.

#### Primary objective

To test the hypothesis that the inclusion of the Salurate pregnancy remote self-monitoring system in pregnant women’s care pathway would help to predict the subsequent development of HDP prior to what is currently possible using traditional methods.

#### Secondary objectives

1. To assess whether Salurate could detect the occurrence of other placentally mediated adverse pregnancy outcomes such as fetal growth restriction (FGR), small for gestational age (SGA) birth, spontaneous preterm birth (sPTB), stillbirth and gestational diabetes mellitus (GDM).
2. To assess the cost-effectiveness of Salurate testing compared with current care methods.
3. To assess the degree of compliance of pregnant women to a self-sampling weekly Salurate test regimen.
4. To assess the algorithm efficacy in a demographically diverse population.

### Salurate system

**Figure 1:**
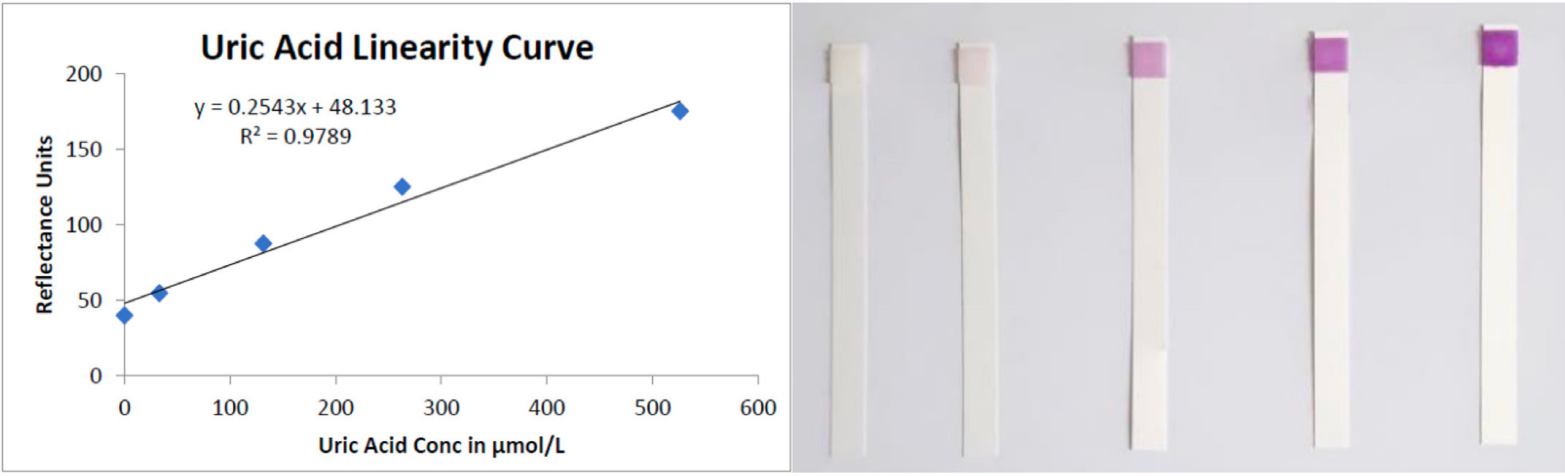
Relationship of colourimetric test to uric acid concentration.

Salurate is a non-invasive, home-use self-sampling system. A saliva sample is applied to colourimetric test paper (encapsulated in a test cartridge). An enzymatic reaction occurs, which produces a purple colour of varying intensities. The intensity of the colour change is directly proportional to the salivary uric acid concentration. While the colour change on the test paper may be visible to the naked eye, accurately interpreting the results is not possible through visual observation alone. The Salurate prediction algorithm employs advanced image analysis techniques to evaluate images submitted over the past few weeks before generating a prediction (figure 1).

**Figure 2:**
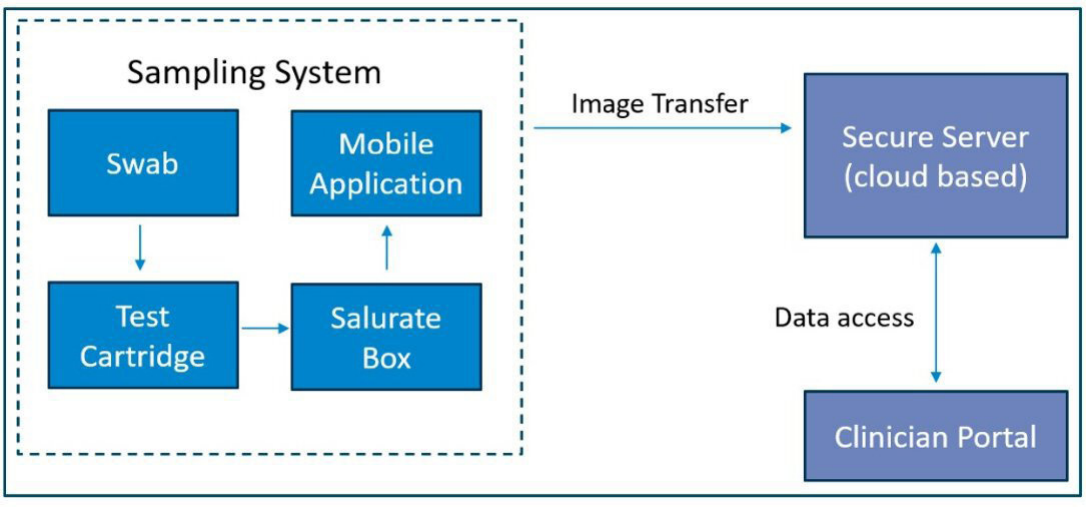
Components of the salurate system.

**Figure 3:**
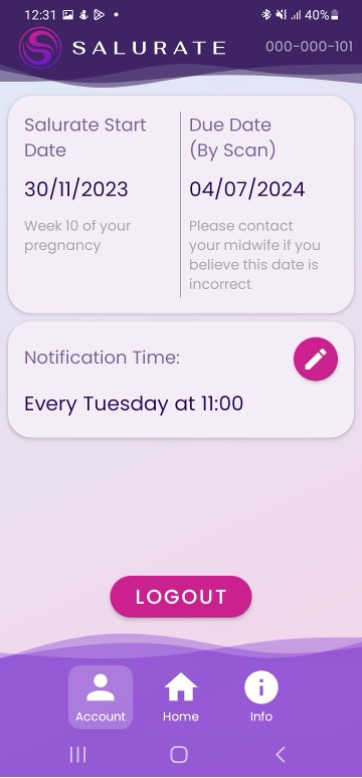
Salurate mobile application.

Components (figure 2):

1. The swab: a prepackaged, sterile swab commonly used for saliva collection. Single use and manufactured from a non-hazardous polyurethane foam.
2. Test cartridge: a bespoke enzymatic uricase colourimetric test assay. The intensity of the purple colour formed is directly proportional to the concentration of uric acid in the saliva sample. The test paper is encapsulated into a single-use test cartridge.
3. Salurate box: Contains all the equipment required to take a sample. The top of the box includes a designated test cartridge area for the purpose of facilitating image capture. The box top is also labelled with the study identification (ID) in alphanumeric and QR code and includes a red/green/blue (RGB) colour chart. The colour chart is used to adjust for differing lighting conditions and adjust for a wide range of camera phone hardware.
4. Mobile application (figure 3): ‘The Salurate app’ facilitates the photographing of colourimetric test paper (test cartridge), guides the participant through the sampling process, transmits the image to the Salurate Secure Server, reminds the participants when to take their sample and provides a source of information about the study.
5. Salurate secure server: a cloud-based server system that utilises the AWS platform. Primary functions:
  1. To receive images transmitted from the Salurate app.
  2. To identify and extract colourimetric data from these images, and to generate predictions based on this extracted data.
  3. To act as a database of test kits, storing information including (but not limited to anonymised participant ID number, test kit batch information, inventory and location, and information relating to recruitment centres and recruiters.
  4. To control and issue notifications to participants and recruiters.
6. Clinical portal: a means of viewing, updating and extracting data from the Salurate secure server. Primary functions:
  1. To facilitate the registering of test kits during the recruitment process.
  2. To facilitate data input on the case report form (CRF).
  3. To facilitate the control of inventory.
  4. To allow low and high-level administrative access to data.

Using the Salurate system:

Participants will be sent a reminder through the Salurate mobile application on a weekly basis to take a saliva swab and upload a photo of the result.

**Figure 4:**
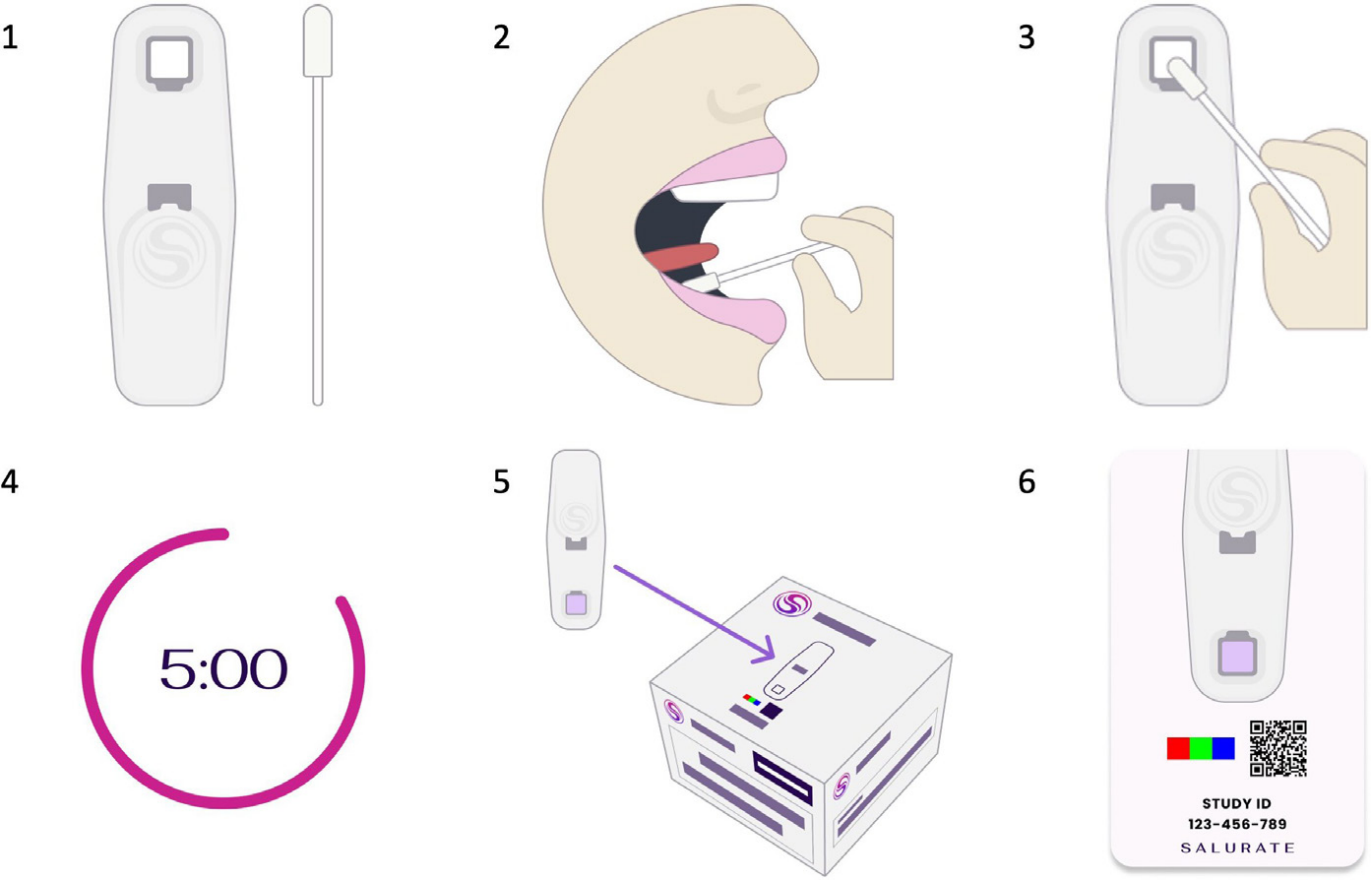
Steps of obtaining a saliva sample.

Steps of obtaining a sample (figure 4):

1. Prepare one test cartridge and one swab, open the Salurate app and select ‘TAKE SAMPLE’.
2. Place the swab under the tongue and leave it there for 30 s.
3. Press the swab into the testing area of the test cartridge and hold it there for 3 s.
4. Let the cartridge sit for 5 min.
5. Place the test cartridge on top of the Salurate box in a well-lit area and take a picture of the testing area, ensuring the colour chart and Study ID number are visible in the photo.
6. Submit the photo to the server using the Salurate mobile application.

## Methods

We used the Standard Protocol Items: Recommendations for Interventional Trials checklist when writing our report.^16^

### Recruitment and sampling

**Figure 5:**
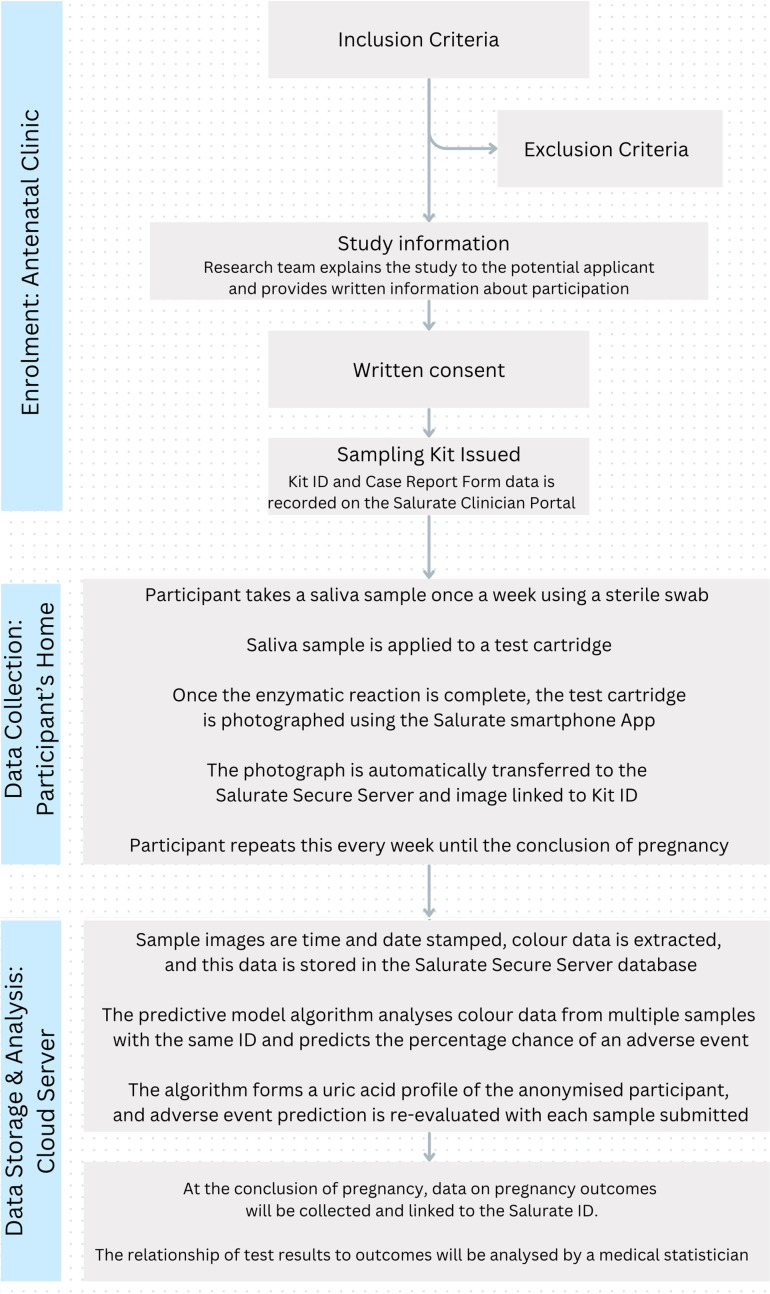
Study schema outlining recruitment, data collection and data analysis.

This is a prospective, multicentre, observational cohort study spanning two trusts across three sites in south-west London: St George’s Hospital NHS Foundation Trust, Epsom and St Helier University Hospitals NHS Trust. The study schema is shown in figure 5. Women attending their first trimester nuchal scan will have a calculation of their risk of hypertensive disease in pregnancy as per the first trimester combined pre-eclampsia screening algorithm.^17^ If their risk of preterm pre-eclampsia is calculated as greater than 1:300 and they satisfy the remaining inclusion criteria, they will be approached and recruited to undertake weekly remote salivary uric acid testing using the Salurate system until the conclusion of pregnancy, with written consent obtained. Participation involves no additional appointments or travel but could extend the initial clinic visit by up to 30 min. Data on antenatal and perinatal maternal and neonatal outcomes will be collected, and the relationship of test results to outcomes will be analysed by a medical statistician. The study is expected to open in September 2024, will be open to recruitment until December 2025 and will close in June 2026.

### Sample size

A sample size of 4000 women was determined after taking into account the number of births at study sites, inclusion and exclusion criteria, and a prevalence of around 11.5% of the events of interest (hypertensive disease in pregnancy, SGA or FGR, gestational diabetes) within the study cohort. Additionally, a sample size of 4000 would allow sensitivity of the Salurate algorithm to identify any of the events of interest with a 95% CI to within plus or minus 5% of the point estimate if sensitivity is found to be around 50%, improving to plus or minus 3% at a higher sensitivity of 90%. Specificity will be estimated to a much higher degree of precision because the proportion of participants that will not encounter adverse outcomes is expected to be much larger.

### Inclusion and exclusion criteria

All women aged 16 years old and over with a viable singleton intrauterine pregnancy between 10 and 16 weeks’ gestation at the time of enrolment, a risk of preterm pre-eclampsia of >1:300 as determined by the Aspirin for Evidence-Based Pre-eclampsia Prevention algorithm^5^ and able to provide informed consent will be eligible for recruitment. The algorithm combines maternal factors, mean arterial pressure (MAP), uterine artery pulsatility index (UtA-PI) and maternal serum pregnancy-associated plasma protein-A (PAPP-A) and PlGF at 11–13 weeks’ gestation and is used routinely at recruiting centres. Exclusion criteria: significant comorbidities that will interfere with participation or physical incapacitation from performing self-sampling; presence of serious mental illness, learning disabilities or educational status or language barrier that influences capacity to use the Salurate app or understand the instructions for use; lack of access to a smartphone or internet at home; periodontal disease, gingivitis or oral cancer.

### Schedule of events

Participants will be approached at their initial visit, and subject to eligibility and consent, will be enrolled in the study. They will be issued a Salurate sampling kit, and a member of the research team will guide the participant through the process of taking a sample and using the Salurate app to upload a picture of their test. Their unique study ID will be registered on the Salurate Clinician Portal and an initial CRF will be completed, recording data on maternal demographics, medical history, obstetric history and data from their first trimester combined pre-eclampsia screening (MAP, PAPP-A, crown-rump length, left and right UtA-PI and risk of preterm pre-eclampsia).

Each participant will submit weekly samples from enrolment until the conclusion of pregnancy. A predictive model algorithm analyses the data supplied by the participants via the Salurate app and provides a prediction with the likelihood of an adverse event (hypertensive disease of pregnancy, FGR) occurring. Several colourimetric measurements are extracted from the uploaded test from the participants, which relate to the concentration of uric acid in the saliva sample. The algorithm looks at the change from week to week, and using the trend from preceding weeks calculates a risk of HDP within a given timeframe. The trend is based on specific patterns of uric acid levels linked with known HDP events learnt from previous trials data. This prediction is calculated post hoc and not revealed to the participant or clinical trial research team. Data on the primary and secondary outcomes will be retrieved from the participants’ medical notes at the conclusion of pregnancy and added to the CRF.

### Outcomes

The study’s primary outcome is the incidence of hypertensive disease of pregnancy. This is defined as any of the following:

- Gestational hypertension.
- Pre-eclampsia.
- Eclampsia.
- Haemolysis, elevated liver enzymes, low platelets syndrome.
- Chronic hypertension with superimposed pre-eclampsia.^18^

Diagnostic criteria are specified in online supplemental material.

Secondary outcomes: SGA (birth weight less than 10th percentile for that gestational age, or 2 SD below the population mean on growth charts); FGR (SGA or birth weight below expected for trajectory of growth with evidence of placental insufficiency)^19^; GDM (any degree of glucose intolerance with onset or first recognition during second or third trimester of pregnancy); sPTB; stillbirth; sampling adherence (frequency of missed samples, dropout rate). Centiles will be determined using Intergrowth references.^20^

### Monitoring

A trial steering committee, comprising an independent chair, chief investigator and lay person stakeholder, will meet at a minimum biannually to discuss trial monitoring. Adverse events and protocol deviations will be reported to the local research team and to the trial sponsors. Protocol amendments will be communicated to all relevant parties.

### Health economics analysis plan

Health economics analysis will be performed by Health Tech Enterprise (HTE) Company Registration Number 5285665. HTE will perform a scoping review, conduct interviews with obstetricians, parameterise the decision analytical model and evaluate the health economic impact of the intervention by assessing the incremental changes as compared against the current care pathway for the categorisation and treatment of patients at risk of pre-eclampsia.

### Data analysis

The quantitative data for this study will be analysed and reported according to the relevant reporting guidelines.^16 21^ All analyses will be performed in StataSE V.16.1 statistical analysis software. All clinical primary and secondary outcomes (maternal and neonatal) will be presented across the whole cohort as percentages with 95% CIs. Missing outcomes data will be presented. Women’s characteristics at enrolment into the study will also be presented with percentages or summary statistics as appropriate.

Salivary uric acid concentration (Salurate) observations will be presented graphically over time from the initial 10–14 weeks pregnancy record through to the end of study participation (1) for the whole cohort, (2) by primary outcome (HDP present or absent) and (3) for each secondary outcome, including adverse maternal or perinatal outcomes. Numbers and percentages of missing Salurate reports will be presented by week of pregnancy with the number of women participating in the study as the denominator (which may vary in the last weeks of the study depending on when they give birth). This will be used to assess the degree of compliance of women to the testing regimen. No multiple imputation or other forms of missing data replacement will be used to estimate missing values of Salurate. No missing data replacement will be employed for clinical outcomes or participant’s characteristics at enrolment, and every effort will be made to record these data. We anticipate that rates of data missingness will be low.

A mixed-methods analysis of longitudinal data will be used to determine the relationship between Salurate results and the primary and secondary outcomes in this cohort. The Salurate algorithm will be employed, and its sensitivity, specificity, PPV and NPV to identify the primary outcome will be presented with 95% CIs. We will also determine how soon the primary outcome would be indicated using Salurate results and compare this with traditional prediction methods such as the FMF algorithm or National Institute for Health and Care Excellence (NICE) checklist. Subgroup analyses will explore whether the Salurate algorithm has greater diagnostic value in particular women according to baseline characteristics, although these analyses may not have enough statistical power to be definitive.

### Patient and public involvement

A range of patient and public involvement activities including surveys and focus groups with women and clinicians have guided choice of participant population, software and test device design. Specific input has been provided by the Action on Pre-eclampsia Charity (APEC). Patient Faced documents were shared with APEC members (consent forms, information leaflets and device designs) and feedback was provided by the service users approached by the charity.

## Ethics and dissemination

Approval has been obtained from Cambridge East Research Ethics Committee (REC reference 24/EE/0123), Medicines and Healthcare products Regulatory Agency (CI/2024/0038/GB) and Health Research Authority (IRAS ID 337290). Participants will be allowed adequate time to read the patient information sheet (PIS) and make a decision about participation in the trial. There are no anticipated disadvantages in taking part in the trial, beyond the potential oral discomfort and 5 minutes a week of the participants’ time to obtain the sample and upload the results to the app. Participation in the study does not alter the participants’ schedule of care. Participants will be provided with information on how to contact the research team in the event of questions, adverse events or withdrawal from the study, both via the PIS and the Salurate app, with no implication of withdrawal on participants’ clinical care.

The results of this study will be published in peer-reviewed journals and presented at national and international conferences. The data obtained by this study will contribute to the design of future studies prior to the approval for clinical use.

## Supplementary material

10.1136/bmjopen-2024-094421 online supplemental file 1
