# Supplementary material for: Validation of salivary uric acid remote self-monitoring for early prediction of hypertensive disorders of pregnancy: study protocol for a prospective, observational, multicentre cohort study
Source: BMJ Open. 2025 Jun 18;15(6):e094421. doi: 10.1136/bmjopen-2024-094421 (PMC12182125; doi:10.1136/bmjopen-2024-094421)
Supplement: online supplemental file 1 [file bmjopen-15-6-s001.docx]

**Supplementary material**

Definitions of hypertensive disorders of pregnancy:

Gestational hypertension

- New hypertension presenting after 20 weeks without significant proteinuria);

Preeclampsia

- New onset hypertension after 20 weeks of pregnancy and coexistence of 1 or more of the following new-onset conditions:
  - Renal insufficiency (creatinine 90 micromol/litre or more
  - Liver involvement (elevated transaminases with or without abdominal pain)
  - Neurological complications (eclampsia, altered mental status, blindness, stroke, clonus, severe headaches, visual disturbance)
  - Haematological complications (platelet count below 150,000/microlitre, disseminated intravascular coagulation or haemolysis)
  - Uteroplacental dysfunction (fetal growth restriction, abnormal fetal dopplers, stillbirth)

Eclampsia

- The occurrence of one or more seizures in a woman with preeclampsia

HELLP syndrome

- Haemolysis, elevated liver enzymes, low platelets

Chronic hypertension with superimposed preeclampsia, either:

- New-onset proteinuria (≥300 mg/24 h) in a woman with hypertension but no proteinuria before 20 weeks' gestation
- A sudden increase in proteinuria or BP, or a platelet count of less than 100,000/mm3, in a woman with hypertension and proteinuria before 20 weeks' gestation
