# Supplementary material for: Validation of salivary uric acid remote self-monitoring for early prediction of hypertensive disorders of pregnancy: study protocol for a prospective, observational, multicentre cohort study
Source: BMJ Open. 2025 Jun 18;15(6):e094421. doi: 10.1136/bmjopen-2024-094421 (PMC12182125; doi:10.1136/bmjopen-2024-094421)
Supplement: Supplementary file 2 [file bmjopen-2024-094421.reviewer_comments.pdf]

PEER REVIEW HISTORY

BMJ Open publishes all reviews undertaken for accepted manuscripts. Reviewers are asked to complete a checklist review form and are provided with free text boxes to elaborate on their assessment. These free text comments are reproduced below.

ARTICLE DETAILS

Title (Provisional)

Validation of salivary uric acid remote self-monitoring for early prediction of hypertensive disorders of pregnancy: study protocol for an observational, analytical cohort study

Authors

Chmielewska, Basia; Reading, Isabel; Bhide, Amarnath; Ganapathy, Ramesh; Thilaganathan, Baskaran

---

VERSION 1 - REVIEW

**Reviewer**	**1**
**Name**	**Battarbee, Ashley N**
**Affiliation**	**The University of North Carolina at Chapel Hill School of Medicine**
**Date**	**16-Feb-2025**
**COI**	**None**

In this study protocol, the authors describe an observational cohort study aimed at validating salivary uric acid remote self-monitoring for early prediction of hypertensive disorders of pregnancy. The manuscript could be improved by addressing the following:

1. Consider adding the word "prospective" to the title, abstract, and discussion of the study design in body of manuscript to clarify that this observational cohort study is prospective and not retrospective.

2. Please define all abbreviations the first time they are used (i.e. FMF, sFlt-1, PlGF in 1st paragraph of introduction, ASPRE in methods section, etc)

3. Please provide details about what criteria are included in the ASPRE algorithm given that this is a major inclusion criteria for the study and will help readers understand who is the target population

4. Please provide definitions of the primary outcome in main body of manuscript or directly reference the supplementary material provided. It should be clear to readers what criteria

are used to diagnose gestational hypertension, preeclampsia, eclampsia, HELLP, superimposed preeclampsia when reading the methods section.

5. Correct typo in introduction (should be HDP and not HPD).

**Reviewer**	**2**
**Name**	**Oros, Daniel**
**Affiliation**	**Universidad de Zaragoza**
**Date**	**09-Apr-2025**
**COI**	**None**

This multi-centre observational cohort study protocol outlines a compelling investigation into the potential of remote salivary uric acid testing, utilising the Salurate method, as a novel tool for the early identification of hypertensive diseases of pregnancy (HDP), including gestational hypertension and preeclampsia. By focusing on a cohort of women identified as being at elevated risk based on first-trimester screening, this protocol offers a rigorous framework for evaluating the clinical utility of this innovative, non-invasive approach in facilitating timely interventions and ultimately improving pregnancy outcomes.

Introduction

Given the authors' established and profound understanding of the complexities inherent in preeclampsia and related hypertensive disorders – as evidenced by their prior contributions to the field – this meticulously designed study holds considerable promise for advancing risk stratification strategies in early pregnancy.

Objectives

The primary objective of this study is to rigorously evaluate the Salurate remote self-monitoring system's ability to predict the development of hypertensive diseases of pregnancy earlier than current standard methods.

Secondary objectives include exploring Salurate's potential in predicting other placentally-mediated adverse pregnancy outcomes, assessing its cost-effectiveness, determining patient compliance with the weekly testing regimen, and evaluating the algorithm's efficacy across a diverse demographic.

This is a well-defined and ambitious set of objectives that directly addresses the potential clinical utility and broader implications of the Salurate system. The inclusion of secondary outcomes and considerations of cost-effectiveness and patient compliance strengthens the overall impact and translational potential of this research.

Methods

The Salurate system is an innovative, non-invasive, home-based salivary uric acid test for early HDP risk prediction. It uses a colorimetric test, a smartphone app for image capture and transmission, a secure server for analysis via a predictive algorithm, and a clinical portal for data management. Its user-friendly design and potential for frequent, remote monitoring offer significant advantages for early risk stratification and proactive pregnancy care.

Here are the potential areas for improvement

Algorithm Specifics: While the analysis plan mentions employing the "Salurate algorithm" and assessing its performance (sensitivity, specificity, PPV, NPV), the underlying logic and the specific variables beyond salivary uric acid it incorporates still remain unclear. Even a general description of the algorithmic approach would be beneficial (e.g., is it based on rate of change, absolute values, specific patterns over time?).

Handling of Confounding Factors: The analysis plan mentions regression models, which allows for the potential adjustment for confounders. However, the protocol does not explicitly list potential confounding factors that will be considered in the analysis (e.g., maternal age, BMI, ethnicity, previous pregnancy history beyond preeclampsia risk).

Longitudinal Analysis Details: While graphical presentation of Salurate data over time is planned, more detail on the specific methods for analysing these longitudinal patterns (beyond the algorithm's output) would be valuable. For example, will time-series analysis techniques be employed?

Definition of "Early Prediction": The primary objective mentions predicting HDP "prior to what is currently possible." The analysis plan states a comparison with "traditional prediction methods using standard NHS monitoring data." However, the specific NHS monitoring data and the timeframe considered "currently possible" for prediction are not explicitly defined. This would be important for contextualizing the Salurate system's potential advantage.

Economic Evaluation Plan: There is no detail provided on the methodology for the cost-effectiveness analysis.

Outline of Statistical Analysis Plan (SAP): While the statistical software and some methods are mentioned, a more structured outline of the SAP, including how specific outcomes will be modelled and how missing data will be handled (beyond the statement of no imputation), would increase transparency.

Clarity on "Traditional Prediction Methods": Explicitly stating which "traditional prediction methods" will be used for comparison and the typical gestational window for their predictive value would provide a clearer benchmark for evaluating Salurate's performance.

Data Quality Control Procedures: While efforts to minimise missing data are mentioned, a brief overview of the data quality control procedures that will be implemented throughout the study would be reassuring.

Clarity on preeclampsia definition and ultrasound references ranges used: The protocol should explicitly detail the diagnostic criteria for the primary outcome of hypertensive diseases of pregnancy, particularly preeclampsia. Given the evolving nature and subtle variations in diagnostic guidelines across different international societies (e.g., ACOG, NICE, ISSHP), clearly stating the specific criteria that will be used in this study is essential for ensuring consistency and comparability of the findings with other research. Furthermore, as the study includes secondary outcomes related to fetal growth restriction (FGR) and utilizes first-trimester Doppler examinations for preeclampsia risk assessment, it is imperative that the protocol specifies the reference ranges that will be employed for fetal weight centiles and uterine artery pulsatility index (PI) measurements. Different reference ranges can lead to variations in the classification of SGA/FGR and the interpretation of Doppler findings. Therefore, clearly stating the chosen reference ranges and their source will enhance the transparency and reproducibility of the study results.

Including these details will significantly strengthen the methodological rigor of the study and ensure that the findings can be accurately interpreted and compared within the broader body of research on hypertensive diseases of pregnancy. Despite these suggestions for refinement, the study protocol presents an excellent and innovative approach to a critical area of obstetric care. The underlying idea and the potential of the Salurate system to improve early risk stratification for hypertensive diseases of pregnancy are highly commendable. Congratulations to the authors for developing such a promising and well-structured research plan.

**Reviewer**	**3**
**Name**	**Di Mascio, Daniele**
**Affiliation**	**University of Rome La Sapienza**
**Date**	**14-Apr-2025**
**COI**	**I confirm I co-authored a couple of papers with Dr Thilanagathan, but all of them in collaborative groups without a direct personal collaboration between the two of us.**

This is a well-structured and well-written study protocol describing a prospective, multicenter cohort study aiming to validate a novel remote, non-invasive salivary uric acid self-monitoring system for early prediction of hypertensive disorders of pregnancy (HDP), which indeed might represent a promising and innovative approach in the field of remote antenatal pregnancy care.

The study design is robust and the methodology is clear. The sample size calculation of 4000 patients in powered for the primary outcome and significantly higher compared to what already published in literature on this topic.

I have a couple of comments/curiosity regarding the sampling which is not well specified in the text:

1. Some salivary test might influenced by external confounders: did patients receive specific instruction regarding when to do the test? i.e. in the morning before eating/brushing teeth/smoking?

2. Was a kind of correlation between the salurate system and serum uric acid ever assessed?

3. Methodology reports that no imputation will be performed and expect low levels of missing data which is understandable for a protocol paper, but I wonder what constitutes acceptable missingness for inclusion in final analysis? Will complete case analysis be used? To me this is notable for a study relying on weekly self-sampling.

4. Can authors anticipate which subgroups (i.e. maternal age, ethnicity, parity) will they focus on and whether any adjustments for multiple comparisons are planned?

Thanks for the opportunity of being a reviewer

VERSION 1 - AUTHOR RESPONSE

Reviewer: 1
Dr. Ashley N Battarbee, The University of North Carolina at Chapel Hill School of Medicine
Comments to the Author:
In this study protocol, the authors describe an observational cohort study aimed at validating salivary uric acid remote self-monitoring for early prediction of hypertensive disorders of pregnancy. The manuscript could be improved by addressing the following:
1. Consider adding the word "prospective" to the title, abstract, and discussion of the study design in body of manuscript to clarify that this observational cohort study is prospective and not retrospective.
Author response: Prospective has been added to title and methods.
2. Please define all abbreviations the first time they are used (i.e. FMF, sFlt-1, PlGF in 1st paragraph of introduction, ASPRE in methods section, etc)
Author response: All abbreviations have now been defined throughout the paper
3. Please provide details about what criteria are included in the ASPRE algorithm given that this is a major inclusion criteria for the study and will help readers understand who is the target population.
Author response: We have added a qualifying sentence to the methods to clarify what is used in the ASPRE algorithm. (235-238)
4. Please provide definitions of the primary outcome in main body of manuscript or directly reference the supplementary material provided. It should be clear to readers what criteria are used to diagnose gestational hypertension, preeclampsia, eclampsia, HELLP, superimposed preeclampsia when reading the methods section.
Author response: We have added a sentence referring directly to the supplementary material. (277)
5. Correct typo in introduction (should be HDP and not HPD). Author response: Corrected.

Thank you for your thoughtful comments!

Reviewer: 2
Dr. Daniel Oros, Universidad de Zaragoza
Comments to the Author:
This multi-centre observational cohort study protocol outlines a compelling investigation into the potential of remote salivary uric acid testing, utilising the Salurate method, as a novel tool for the early identification of hypertensive diseases of pregnancy (HDP), including gestational hypertension and preeclampsia. By focusing on a cohort of women identified as being at elevated risk based on first-trimester screening, this protocol offers a rigorous framework for evaluating the clinical utility of this innovative, non-invasive approach in facilitating timely interventions and ultimately improving pregnancy outcomes.

Introduction
Given the authors' established and profound understanding of the complexities inherent in preeclampsia and related hypertensive disorders – as evidenced by their prior contributions to the field – this meticulously designed study holds considerable promise for advancing risk stratification strategies in early pregnancy.

Objectives
The primary objective of this study is to rigorously evaluate the Salurate remote self-monitoring system's ability to predict the development of hypertensive diseases of pregnancy earlier than current standard methods.
Secondary objectives include exploring Salurate's potential in predicting other placentally-mediated adverse pregnancy outcomes, assessing its cost-effectiveness, determining patient compliance with the weekly testing regimen, and evaluating the algorithm's efficacy across a diverse demographic.
This is a well-defined and ambitious set of objectives that directly addresses the potential clinical utility and broader implications of the Salurate system. The inclusion of secondary outcomes and considerations of cost-effectiveness and patient compliance strengthens the overall impact and translational potential of this research.

Methods
The Salurate system is an innovative, non-invasive, home-based salivary uric acid test for early HDP risk prediction. It uses a colorimetric test, a smartphone app for image capture and transmission, a secure server for analysis via a predictive algorithm, and a clinical portal for data management. Its user-friendly design and potential for frequent, remote monitoring offer significant advantages for early risk stratification and proactive pregnancy care.

Here are the potential areas for improvement
Algorithm Specifics: While the analysis plan mentions employing the "Salurate algorithm" and assessing its performance (sensitivity, specificity, PPV, NPV), the underlying logic and the specific variables beyond salivary uric acid it incorporates still remain unclear. Even a general description of the algorithmic approach would be

beneficial (e.g., is it based on rate of change, absolute values, specific patterns over time?).
Author response: Several colorimetric measurements are extracted from the uploaded test from the participants, which relate to the concentration of uric acid in the saliva sample. The algorithm looks at the change from week to week, and using the trend from preceding weeks calculates a risk of HDP within a given timeframe. The trend is based on a specific patterns of uric acid levels linked with known HDP events learnt from a previous trials data. This explanation has been included (264-269)
Handling of Confounding Factors: The analysis plan mentions regression models, which allows for the potential adjustment for confounders. However, the protocol does not explicitly list potential confounding factors that will be considered in the analysis (e.g., maternal age, BMI, ethnicity, previous pregnancy history beyond preeclampsia risk).
We will consider all known biophysical and demographic characteristics that are associated with pre-eclampsia, i.e. age, BMI (continuous), parity, ethnicity, obstetric history, medical history (categorical). These will be considered as confounders and will also be used in the development of a multipile regression algorithm to predict onset of pre-eclampsia. This is referenced in lines 334-337.
Longitudinal Analysis Details: While graphical presentation of Salurate data over time is planned, more detail on the specific methods for analysing these longitudinal patterns (beyond the algorithm's output) would be valuable. For example, will time-series analysis techniques be employed?
We will use mixed methods analysis of longitudinal data to predict onset of PE and related, placentally-mediated outcomes. This is reflected under data analysis (328).
Definition of "Early Prediction": The primary objective mentions predicting HDP "prior to what is currently possible." The analysis plan states a comparison with "traditional prediction methods using standard NHS monitoring data." However, the specific NHS monitoring data and the timeframe considered "currently possible" for prediction are not explicitly defined. This would be important for contextualizing the Salurate system's potential advantage.
Author response: Thank you for identifying this. Currently, within the NHS, women are classified into high or low risk for pre-eclampsia, and subsequently recommended Aspirin, and all women have blood pressure and urine assessment at antenatal visits. Therefore, categorising risk throughout pregnancy is static and we rely on clinical signs and symptoms to manifest to know that the woman is affected by HDP. Incorporating the salurate system would allow for a dynamic risk assessment throughout pregnancy, and highlight women at a higher risk of developing HDP within e.g. the next 4 weeks, and allow for tailored antenatal care e.g. more frequent blood pressure monitoring, assessment of fetal wellbeing. This will be clarified in the health economics analysis.
Economic Evaluation Plan: There is no detail provided on the methodology for the cost-effectiveness analysis.
Health economics analysis is now detailed from line 219.
Clarity on "Traditional Prediction Methods": Explicitly stating which "traditional prediction methods" will be used for comparison and the typical gestational window for their predictive value would provide a clearer benchmark for evaluating Salurate's performance.

Author response: This has been clarified to be the first trimester ASPRE/FMF algorithm and/or NICE checklist risk prediction which is currently used in the NHS.

Data Quality Control Procedures: While efforts to minimise missing data are mentioned, a brief overview of the data quality control procedures that will be implemented throughout the study would be reassuring.

Author response: We anticipate that there will be some missing data as participants may forget, be travelling or hospitalised, and we will need to determine a threshold for acceptability of missing data. In previous studies, data in missing weeks was substituted with data from the previous week

Clarity on preeclampsia definition and ultrasound references ranges used: The protocol should explicitly detail the diagnostic criteria for the primary outcome of hypertensive diseases of pregnancy, particularly preeclampsia. Given the evolving nature and subtle variations in diagnostic guidelines across different international societies (e.g., ACOG, NICE, ISSHP), clearly stating the specific criteria that will be used in this study is essential for ensuring consistency and comparability of the findings with other research. Furthermore, as the study includes secondary outcomes related to fetal growth restriction (FGR) and utilizes first-trimester Doppler examinations for preeclampsia risk assessment, it is imperative that the protocol specifies the reference ranges that will be employed for fetal weight centiles and uterine artery pulsatility index (PI) measurements. Different reference ranges can lead to variations in the classification of SGA/FGR and the interpretation of Doppler findings. Therefore, clearly stating the chosen reference ranges and their source will enhance the transparency and reproducibility of the study results.

Author response: Definitions for the primary outcomes have been specified in supplementary material, which has now been explicitly referenced in the main text (ISSHP). This has been updated to include definitions of FGR. We have specified the reference ranges that will be used for fetal, birthweight and doppler centiles.

Including these details will significantly strengthen the methodological rigor of the study and ensure that the findings can be accurately interpreted and compared within the broader body of research on hypertensive diseases of pregnancy. Despite these suggestions for refinement, the study protocol presents an excellent and innovative approach to a critical area of obstetric care. The underlying idea and the potential of the Salurate system to improve early risk stratification for hypertensive diseases of pregnancy are highly commendable. Congratulations to the authors for developing such a promising and well-structured research plan.

Author response: Thank you for your helpful and in-depth comments, which have prompted further thought and clarification.

Reviewer: 3

Dr. Daniele Di Mascio, University of Rome La Sapienza

Comments to the Author:

This is a well-structured and well-written study protocol describing a prospective, multicenter cohort study aiming to validate a novel remote, non-invasive salivary uric acid self-monitoring system for early prediction of hypertensive disorders of pregnancy (HDP), which indeed might represent a promising and innovative approach in the field of remote antenatal pregnancy care.

The study design is robust and the methodology is clear. The sample size calculation of 4000 patients in powered for the primary outcome and significantly higher compared to what already published in literature on this topic.

Author response: Thank you for your thoughtful comments.
I have a couple of comments/curiosity regarding the sampling which is not well specified in the text:
1. Some salivary test might influenced by external confounders: did patients receive specific instruction regarding when to do the test? i.e. in the morning before eating/brushing teeth/smoking?
Author response: Participants are asked not to eat or drink for 30 minutes before doing the test, as such, we suggest to them to do the test first thing in the morning, and indeed before brushing teeth. We are collecting data on whether or not participants smoke cigarettes, and will look at whether this affects the system's efficacy.
2. Was a kind of correlation between the salurate system and serum uric acid ever assessed?
Author response: There have been studies that have correlated uric acid levels in serum and saliva (e.g. https://doi.org/10.1016/j.clinbiochem.2011.09.012, DOI: 10.7759/cureus.48457) and a previous study (Puschl. Et al) showed correlation between the salurate system and serum uric acid.
3. Methodology reports that no imputation will be performed and expect low levels of missing data which is understandable for a protocol paper, but I wonder what constitutes acceptable missingness for inclusion in final analysis? Will complete case analysis be used? To me this is notable for a study relying on weekly self-sampling.
Author response: We anticipate that there will be some missing data as participants may forget, be travelling or hospitalised, and we will need to determine a threshold for acceptability of missing data. In previous studies, data in missing weeks was substituted with data from the previous week
4. Can authors anticipate which subgroups (i.e. maternal age, ethnicity, parity) will they focus on and whether any adjustments for multiple comparisons are planned?
Author response: During data analysis we will look at whether the algorithm performs similarly across ethnic subgroups. Maternal characteristics may be incorporated to improve the efficacy of the predictive algorithm.
